# Food Waste Governance Architectures in Europe: Actors, Steering Modes, and Harmonization Trends

**DOI:** 10.1002/gch2.202300265

**Published:** 2024-08-08

**Authors:** Julia Szulecka, Carrie Bradshaw, Ludovica Principato

**Affiliations:** ^1^ Norwegian Institute for Water Research (NIVA) Økernveien 94 Oslo 0579 Norway; ^2^ University of Leeds Leeds LS2 9JT UK; ^3^ Department of Business Economics Roma Tre University Via Silvio D'Amico, 77 Rome 00145 Italy

**Keywords:** food waste governance, food waste law, reduction targets, SDG, voluntary agreements

## Abstract

The scale of food waste across Europe is alarming, and its reduction is becoming an important public policy and governance issue. Sustainable Development Goal Target 12.3 constitutes a global attempt to galvanize system‐level reductions. In response, layers of varied regulation and governance at regional and national levels have emerged. This paper studies the types of governance architectures visible across Europe, what policy interventions they bring, and whether responses to the food waste challenge are converging. It looks at four leading food waste jurisdictions—France, England, Norway, and Italy—and investigates the hidden realities obscured by the simplistic division of legislative/top‐down versus voluntary/bottom‐up approaches. It applies a governance matrix to understand the variety of food waste “steering modes”, exploring both the extent to which a regime is hierarchical and/or non‐hierarchical and why. Notably, the paper also identifies some general tendencies in food waste governance, including legislative threats, challenges in distributing responsibility across the actors, focus on “low hanging fruits”, and an overall harmonization of policy responses in a neoliberal paradigm, with redistribution often pursued as a panacea for the food waste crisis.

## Graphical abstract

Food waste reduction is becoming an important public policy and governance issue in Europe. This paper looks at four countries that approach food waste reduction differently—France, England, Norway, and Italy—and investigates what actual governance approaches hide beneath the division between ‘legal’ and ‘voluntary’ approaches. It maps the differences and similarities between the four cases and identifies some general tendencies in food waste governance.

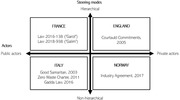

## 1 Introduction

Food waste is receiving increasing attention in both scholarship and policy circles. Its root causes can be linked to concrete stages in the food supply chain (agricultural production, post‐harvest handling and storage, processing and packaging, distribution, and finally – consumption) and their interactions. Typical causes of food waste and loss differ within the stages of the food value chain and further depend on economic development, climate, culture, diet, or popular food products. For agricultural production, typical causes are spillage and quality standards, agricultural policies, and labor costs. For post‐harvest handling it is also spillages, degradation, interruptions, and food produce damage. Processing, packaging, and transport bring degradation, market rejections and spillage. In distribution, food waste is caused by legal restrictions and aesthetic requirements. At the consumption stage, typical causes are poor planning, oversized portions, lack of awareness and understanding of date marking, and bulk purchases.^[^ ^1^ ^]^ The problem cannot be solved with mere incremental change and cannot be fully addressed by the actors individually; it requires system‐level reductions and collaboration within the food value chains, and targets have emerged as a fundamental governance tool at the international, regional, and national level to galvanize action for food waste prevention.

Importantly, Sustainable Development Goal (SDG) Target 12.3 provides that per capita global food waste at retail and consumer levels should be halved by 2030, with reductions in food losses along production and supply chains (including post‐harvest losses).^[^ ^2^ ^]^ Although there is a broad consensus to prevent food waste in line with the SDG 12.3 target, and legally binding targets are also expected, e.g., at the European Union level,^[^ ^3^ ^]^ the work toward fulfillment of the goal indicates a considerable variation among countries, sectors, and stages in the value chain. The common framework of the so‐called waste hierarchy model defines preferred (prevention and reduction of waste) and less preferred (recycling, recovery, and disposal) waste governance strategies but does not define any concrete governance tools and actors responsibilities.^[^ ^4^ ^]^

The food waste problem “has made a significant climb on governance agendas since 2010”^[^ ^5^ ^]^ and this is equally visible in macro‐targets and in the layers of regional and (sub)national regulation. However, it is often claimed that because of ad‐hoc, fragmented governance arrangements, and limited ambition, national efforts are insufficient to meet the global target.^[^ ^6^, ^7^, ^8^, ^9^, ^10^ ^]^

Meanwhile, the discussion of food waste reduction governance is often one‐dimensional, focused on the mere presence of food waste reduction laws, and countries that introduced them are depicted as more ambitious and receive significant attention as apparent frontrunners. In the media, the first European food waste laws were dubbed as “a war on waste”, “groundbreaking” and bringing “a revolution”.^[^ ^11^, ^12^, ^13^ ^]^ This overly simplistic characterization creates a dichotomy between food waste laws versus their absence. Little attention is paid to more complex dynamics and to what governance architectures actually emerge in different countries to address food waste.

In response, this paper investigates food waste governance by applying a two‐dimensional framework to both the type of interventions adopted, and the regimes that produce them, to capture the *variety* of food waste reduction governance architectures. Crucially, it explores both the extent to which a governance architecture is top‐down (“hierarchical”) and/or bottom‐up (“non‐hierarchical”), but also analyses the actor constellations—explicitly or implicitly—in play across the “public‐private” divide/continuum. We ask *what are the types of governance architectures visible across Europe, what policy interventions do they bring and are responses to the food waste challenge converging?*

We use a two‐dimensional framework drawing from Hall (2011) for a comparative case study analysis of four countries which are often discussed in the food waste reduction and prevention literature as examples of apparently different approaches. France and Italy are widely considered paragons of a “top‐down” approach because of the explicit food waste laws they introduced,^[^ ^14^ ^]^ in contrast to the so‐called “voluntary” approaches in the UK and Norway.^[^ ^15^ ^]^ Our structured comparative analysis shows the extent to which the emergent food waste reduction governance architectures are divergent, but also in which terms they seem to be increasingly aligning and harmonizing over time. Further, we provide a Discussion section, reflecting on the different governance architectures, hidden similarities, and some common trends in food waste reduction governance.

The paper identifies important similarities across cases that are often categorized as divergent in approaches (France and England, as well as Norway and Italy). At the same time, we note some general tendencies in food waste governance, including legislative threats, challenges in distributing responsibility across the actors, focus on “low hanging fruits”, and an overall harmonization of responses in a neoliberal paradigm, with redistribution often pursued as a panacea for the food waste crisis.

## 2 Theory and Method

### 2.1 Governing Food Waste: Steering Modes and Actor Constellations

Capturing the variety of responses to the food waste problem and regulatory measures adopted as attempts to reach the SDG target requires a framework that goes beyond the legislation/no legislation dichotomy. For this, we draw on governance scholarship.

Governance is a “way of organizing collective action”,^[^ ^16^ ^]^ by means of mobilizing various actors and organizing their effective interaction, creating the conditions for taking binding decisions, and expanding the set of actors involved in decision‐making to increase not only effectiveness but also legitimacy. The result is “a system of co‐production of norms and public goods where the co‐producers are different kinds of actors”.^[^ ^17^ ^]^ Consequently, we can distinguish specific *governance architectures*, that is the structures, processes, and mechanisms that guide decision‐making, accountability, and control within an organization or system.^[^ ^18^ ^]^

What is important for our analysis is the way governance theory approaches the frontier between the public and private sectors.^[^ ^19^ ^]^ The move beyond traditional top‐down and centralized steering logically spawns a “hybridization of modes of control that allow the production of fragmented and multidimensional order within the state, by the state, without the state, and beyond the state”.^[^ ^20^ ^]^ Governance scholarship looks at how states lose the capacity for direct control, replacing it with a capacity to influence actors’ behaviour by using a number of less direct forms of intervention.^[^ ^21^, ^22^ ^]^

Strict distinctions between state and non‐state governing are rare and difficult, as states delegate power and such delegation can have many forms, from specialist governmental agencies to ceding competences and resources to local governments. It can also mean engaging with other actors, as well as with public and private entities exchanging and blending resources.^[^ ^21^ ^]^

Food waste could seem like a straightforward problem, with relatively stable value chains and concurring, identifiable market failures. Many causes are very tangible and depend on unforeseen weather shocks, costs related to reducing food waste (at several food chain stages), pricing of external effects related to food waste or good information access. Moreover, there is a universal agreement among actors that wasting food is wrong (morally, economically, and environmentally). A closer look, however, reveals a more “wicked” problem as a range of different actors, and institutions with diverse underlying perspectives drive food waste prevention and reduction tools.^[^ ^23^ ^]^ More ambitious reductions are therefore increasingly perceived as a wicked problem with increased scope and complexity requiring new governance approaches.

In a highly complex society with problems extending across scales and/or borders, central actors—governments—are often unable to obtain the knowledge required to form effective instruments of intervention, making centralized and hierarchical steering extremely difficult or downright impossible. Public actors are “unable to muster the knowledge required to shape effective instruments of intervention” and need to depend on broader expertise and knowledge^[^ ^24^ ^]^ but also on implementation and enforcement.^[^ ^25^ ^]^ Thus, many governments encourage other actors to address environmental problems voluntarily.^[^ ^26^, ^27^, ^28^ ^]^ Self‐governing capacity of decentralized actors is mostly welcome in complex and wicked problems,^[^ ^29^ ^]^ where it is difficult to agree on means, definitions, and evaluation frameworks.^[^ ^30^ ^]^ We can distinguish three potential drivers of self‐regulation: where industrial actors try to prevent governmental intervention with self‐regulatory actions to forestall the legislative threat, where social pressures and anticipated consumer behavior push for certain corporate behavior or from the company's own values and motivation that something must be done.^[^ ^9^, ^31^, ^32^, ^33^ ^]^

Preventing food waste brings public and private sectors together to transfer ideas and develop innovative solutions.^[^ ^34^ ^]^ However, self‐governing spaces are not automatically effective and efficient, and they also create accountability problems.^[^ ^35^ ^]^ Public meta‐governance should strike the right balance between over‐regulation and under‐regulation of self‐governing actors. The first diminishes motivation, and reduces ownership feeling and creativity. The second might lead to avoiding conflicts, promoting the status quo or the lowest common denominator.^[^ ^36^ ^]^

### 2.2 Conceptual Framework, Methodology and Data

Non‐legislative responses to food waste constitute a heterogenous category, and on the other hand, food waste laws can be constructed in very different ways and engage key actors in the sector and along food value chains, a variety which has to be categorized more accurately to enable learning across cases and overall, increase the chances to meet the SDG target.

**Figure 1:**
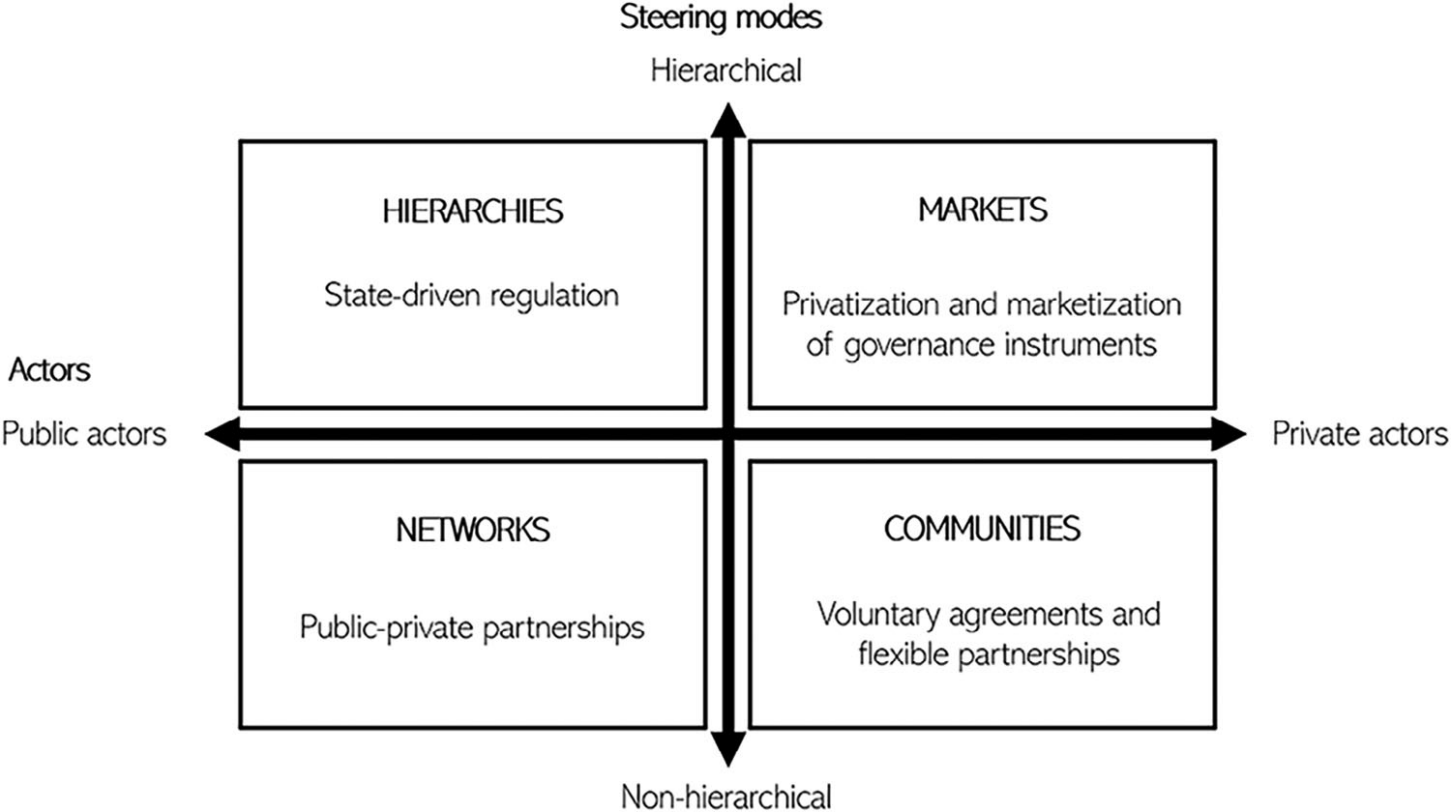
Governance architecture typology (authors’ own elaboration adapted from Hall 2011).

Understanding that contemporary public policy and responses to wicked problems such as food waste cannot be reduced to the distinction between direct state response (in the form of legislation) or lack thereof, we propose a two‐dimensional matrix of governance architectures. We identify two dimensions with actors across the public and private spectrum and steering modes with more hierarchical or non‐hierarchical regimes (see **Figure** **1**).

Those two dimensions have been elaborated by Hall,^[^ ^37^ ^]^ in order to demonstrate and better understand the *variety* of governance architectures, initially applied to study tourism policies. The resulting four ideal‐typical constellations contain elements widely applied in governance studies. Hierarchies, markets, and networks have been presented as three typical modes of coordination already by Frances et al.,^[^ ^38^ ^]^ while Pierre and Peters^[^ ^39^ ^]^ added the fourth category of community.

Each ideal type has some inherent characteristics and promotes different policy instruments. *Hierarchies* strongly distinguish between the public and private policy space, use command and control and hierarchical relationships. Policymakers, legislators, and central government are key actors. *Markets* see market mechanisms as efficient and just regulation and assume consumers as rational actors can make good decisions, based primarily on price signals. Here bargaining takes place between producers and consumers and the policy arena is dominated by economic actors who can take the lead and engage in voluntary norm‐setting if that is consistent with their interests. *Networks*, on the other hand, facilitate coordination between public and private actors, bring more policy bargaining and negotiations with different stakeholder groups, with public actors in the lead. Finally, *Communities* bring more civic spirit into governance processes and a “grassroots” scale, where communities govern with minimal state involvement, in a bottom‐up and largely voluntary manner.

We apply this conceptual framework to a comparative analysis of four country case studies. Our case selection is driven by the regulatory context and national engagement in food waste work and represents the frontrunners in top‐down food waste reduction governance (Italy and France with the first European food waste laws) and bottom‐up approaches to food waste governance (the United Kingdom, UK, more specifically England,^[^ ^40^ ^]^ and Norway with most established voluntary agreements on food waste). All case study countries are internationally perceived as leaders in food waste interventions,^[^ ^5^, ^9^, ^14^ ^]^ even before the issue has been officially addressed by the SDG and European Union (EU) policy agendas.

For each case, we provide (i) a brief historical sketch of the evolution of food waste governance, followed by the two‐dimensional analysis, where (ii) *steering modes* discuss to what extent the hierarchies and hierarchical relationships and arrangements are discernable and (iii) to analyze *actors’ constellations* we map key actors involved in food waste reduction work. In the last category (iv) *focus areas of food waste governance architecture* are discussed. Case‐study analysis draws on the relevant primary food waste law and policy documents analysis, a desktop review of academic scholarship, and grey literature, together with data gathered from the “Building REsponsibility And Developing Innovative Strategies For Tackling Food Waste” (BREAD) project, which included background interviews with food waste experts and stakeholder workshops.

## 3 Analysis

### 3.1 France

#### 3.1.1 Historical Development

France has had an active tax policy encouraging food donations since the 1980s. Tax incentives started with food businesses but were later extended to farmers, processors, and logistic operators. The 2013 National Pact against Food Waste^[^ ^41^, ^42^ ^]^ was followed by two major laws: the Garot Law from 2016 and the Egalim Law from 2018. The former is known as the first European law on food waste and notable for obligating certain retailers (supermarkets of more than 400m^2^) to sign agreements with food redistribution charities to donate surplus food and prohibiting the deliberate destruction of food still fit for human consumption. Further legal measures from 2018 and 2020 require the food industry to measure the amount of food they waste.^[^ ^42^ ^]^

#### 3.1.2 Steering Modes

France is often presented as illustrating a hierarchical or “top‐down” governance approach, exemplified by the ongoing and extensive use of legislation and regulation. The Garot Law is sometimes described as the world's first “punitive” food waste law^[^ ^43^ ^]^ to adopt formally strong and binding language.^[^ ^42^ ^]^ “Command and control” style regulation is deployed, with possible sanctions for non‐compliance with obligations such as those to enter into donation agreements, offer doggy bags and improve donations, together with a ban on the deliberate destruction of surplus food.

However, while widely reported as banning supermarket food waste^[^ ^44^ ^]^ and implementing a “mandatory donation” requirement,^[^ ^45^ ^]^ there is no requirement to donate any minimum quantities/proportions of food (in contrast to a similar approach adopted in California); sanctions have as yet been levied; and there is no capacity in any case for monitoring compliance.^[^ ^42^, ^46^ ^]^ The Garot Law thus promotes the social expectation of donating food, rather than *requiring* it,^[^ ^41^ ^]^ adopting a “suasive” approach relying on “symbolic messaging and voluntary engagement”.^[^ ^42^ ^]^ Furthermore, the (not‐so‐mandatory) donation obligation may be less significant for donations than relatively high tax incentives (60% capped at 0.05% of annual turnover), without which supermarkets would likely send surplus to anaerobic digestion.^[^ ^41^, ^47^, ^48^ ^]^ A reliance on pricing mechanisms is more typically associated with hierarchy through *markets*, with the below analysis of actor constellations further demonstrating how the French approach is somewhat mischaracterized in the literature as the “top‐down” exemplar.

#### 3.1.3 Key Actors

The extensive use of law and regulation places the *state* at the center of food waste governance in France. However, collaborative, multi‐stakeholder workshops alongside a variety of grassroots activities have also played a significant role,  with a variety of actors, including environmental non‐profits, consumer organizations, food assistance organizations, and representatives of the agrifood, retail, and food service sectors.^[^ ^42^, ^45^ ^]^ Notably however, the outcome of these multistakeholder processes was state intervention.

That said, France falls between different governance approaches. By “increasing the capacity of the market”, the Garot Law provides a policy arena for private economic actors to tackle food waste through a more market‐oriented approach. For example, the Garot Law “transformed” surplus food into a resource and thus created business opportunities for a new category of private actors: new for‐profit start‐ups which are increasingly inhabiting a sector previously populated by non‐profit charities.^[^ ^49^ ^]^ Furthermore, while state regulation of food waste is typically associated with a focus on the public or common good, the French approach has actually institutionalized a narrative “that prioritizes profit over social equity”.^[^ ^50^ ^]^ Moreover, a variety of campaigns have sought to place responsibility on consumers for food waste (rather than broader structures),^[^ ^42^ ^]^ further emphasising the role of market actors.

Furthermore, France appears to target sectors of the supply chain individually. The Garot Law initially targeted retailers,^[^ ^51^ ^]^ or more specifically, retail‐level food waste, which may be somewhat misplaced given the relatively small quantities produced at this stage.^[^ ^52^ ^]^ While these obligations have since been extended to caterers, food businesses and wholesale traders, this approach represents a relatively narrow understanding of retail responsibility for food waste, given supermarkets are indirectly responsible for food waste that arises *elsewhere* in the supply chain, through, e.g., purchasing agreements (upstream) or marketing campaigns (downstream).^[^ ^47^, ^53^ ^]^ The Garot Law instead puts responsibilities on retailers only for the waste they generate in retail. Notably, retailers dominated the pre‐legislative policy discussion and blocked potentially more transformative intervention.^[^ ^42^ ^]^

#### 3.1.4 Focus Areas of Food Waste Governance Architecture

The French approach primarily targets the re‐use/redistribution of surplus food. It has thus been widely criticized as treating the symptoms rather than root causes of food waste,^[^ ^42^, ^49^, ^52^, ^54^, ^55^ ^]^ and potentially exacerbating challenges relating to overproduction by perpetuating a norm of abundance while incentivizing retailers to shift food waste elsewhere in the supply chain.^[^ ^42^, ^56^, ^57^ ^]^ Approaches that ensure retailer responsibility for food waste implies the need for holistic interventions that prioritize “strong” prevention at all stages of the supply chain and address power imbalances in the supply chain.^[^ ^54^, ^58^ ^]^ However, despite the recent EU Directive on unfair trading practices (UTPs – practices that grossly deviate from good commercial conduct)^[^ ^59^ ^]^ and alternatives to retailer‐driven food systems, French policies such as mandatory measurement have started to support such alternatives in only marginal ways.^[^ ^42^ ^]^

### 3.2 Italy

#### 3.2.1 Historical Development

Coordinated action against food waste in Italy started with the Good Samaritan Law, passed in 2003. It encouraged restaurant owners to donate uneaten food and debureaucratized food donations, simplifying procedures for non‐profit organizations working on food redistribution. In 2011 the “Zero Waste Charter” was established, an agreement signed by 800 Italian municipalities to implement active policies aimed at reducing food waste at the local and municipal level.^[^ ^60^ ^]^ In 2013, the Ministry of the Environment and the Protection of the Territory and the Sea launched the National Waste Prevention Program opening the door to the first national Food Loss and Waste Prevention Plan, also known as PINPAS, published in 2014. The plan highlights ten priority measures to reduce food waste at different level of the food supply chain, with a special emphasis on surplus donations.^[^ ^61^ ^]^

Finally, with the Gadda Law from 2016, Italy became the second European country after France to implement a legislative system to address the problem of food loss and waste. Through its 18 articles, it regulates different areas of application to reduce food waste, favoring the transformation and redistribution of food surpluses along the entire food supply chain, implementing policies to promote the reuse and recycling of food waste, and supporting research, information, and awareness‐raising activities. It created the National Table for Combating Food Waste, a coordination group in charge of presenting policy proposals, supervising monitoring and evaluation activities, and improving practices. The new measures had a quick effect of increased food donations by 30% in the following year.

#### 3.2.2 Steering Modes

Although its approach is considered as a top‐down one, the Gadda Law does not include mandatory obligations or punitive sanctions, but it is rooted in voluntary agreements that involve different stakeholders. A similar approach is also used at the Italian regional level, where most regions have committed to adopting their own legislation with a focus on promoting food surplus distribution and reducing food waste.^[^ ^62^ ^]^ At a local level, cities have proven to be key actors in preventing food waste as a result of successful policies and initiatives to combat it.^[^ ^63^ ^]^ By acting on an urban scale, cities can combine the regulatory approach with the administrative one, adopting multi‐level and multi‐sector policies. Indeed, the prevention of food waste requires a variety of initiatives that involve various local public authorities, such as cities, provinces, and other actors including citizens, school canteens, non‐governmental organizations, retailers, food markets, and restaurants.^[^ ^64^, ^65^, ^66^, ^67^ ^]^

An example of urban food policy adopted in Italy is the international Milan Urban Food Policy Pact, an agreement signed by 160 cities around the world that binds the authorities of the cities to collaborate to create sustainable food systems, ensure that everyone has access to nutritious meals, protect biodiversity, and combat food loss and waste.

#### 3.2.3 Key actors

Concerning the public and private actors involved, the Gadda Law mainly targets authorities, food industry, end consumers, and non‐governmental organizations (NGOs). Indeed, by filling up some of the holes left by the previous “Good Samaritan” law, the Gadda Law simplifies the donation procedure to boost the number of donors,^[^ ^68^ ^]^ thereby primarily involving food industry and supply chain agreements. Furthermore, municipalities have also been given an important role. They have the authority to give a local waste tax reduction incentive to retailers that donate, and they oversee encouraging the use of doggy bags in restaurants. Finally, given the focus on awareness‐raising campaigns to encourage the prevention of food waste, consumers are also central.

#### 3.2.4 Focus Areas of Food Waste Governance Architecture

While Italy is the exemplar of the top‐down legislative approach to food waste, the resulting governance arrangement is creating food waste reduction networks engaging a large variety of actors. It can be observed that food donation is a key issue in the food waste prevention governance, with procedural, fiscal, and sanitary measures that encourage food donations.

At the national level, since the Gadda Law aims at encouraging the donation and redistribution of food surpluses, the Italian food waste governance focuses on “weak” waste prevention and reuse of products, with a specific focus on redistribution.^[^ ^69^ ^]^ Regional action and most of the initiatives at the local and city level are also mostly targeting food redistribution.^[^ ^69^ ^]^

### 3.3 England

#### 3.3.1 Historical Development

Food waste emerged as a policy issue in 2000 and has since worked its way up the agenda.^[^ ^56^ ^]^ 2005 saw the signing of the Courtauld Commitment, the world's first voluntary agreement to tackle food waste. Later in 2007, government identified food waste as a “priority waste stream”,^[^ ^70^ ^]^ and the first “large‐scale” interventions to tackle food waste commenced, centered around the “Love Food Hate Waste” campaign.^[^ ^71^ ^]^ Courtauld has since expanded in scope, and now provides a series of non‐binding targets aligned with SDG 12.3, alongside the *Food Waste Reduction Roadmap's* “Target Measure Act” approach.^[^ ^72^ ^]^ In 2018, the Government also proposed several hierarchical measures, including mandatory reporting and reduction targets.^[^ ^58^ ^]^ However, at the time of writing, these are yet to be implemented, and it remains to be seen whether England will move away from the markets approach outlined below.

#### 3.3.2 Steering Modes

England represents a hierarchical approach to food waste governance through markets rather than state intervention. Bradshaw^[^ ^58^ ^]^ demonstrates how government “stepped back” from food waste, with regulation as a last resort “hollowing out” the governance space,^[^ ^58^ ^]^ see also.^[^ ^73^ ^]^ Instead, government argued that a more sustainable economy “can and should be delivered with limited government intervention as industry responds to the clear business case” for waste prevention.^[^ ^74^ ^]^

Market approaches typically involve the privatization/corporatization of state functions and the use of voluntary instruments. In England, this is most obviously illustrated by outsourcing the administration of voluntary efforts to the Waste and Resources Action Programme (WRAP).^[^ ^58^ ^]^ Another example is the increasing reliance on third sector/charitable organizations to redistribute surplus food, in turn shifting burdens for preventing waste away from the state.^[^ ^58^, ^75^ ^]^ WRAP has relied in Courtauld on creating a “pre‐competitive” space to support collaboration between private actors, suggesting an aspiration of a non‐hierarchical network approach. However, evidence suggests that competition has “crept in” to these spaces, undermining collaboration and highlighting the overall dominance of the market.^[^ ^76^, ^77^ ^]^

#### 3.3.3 Key Actors

Market approaches typically emphasize *private business actors*, as well as consumers, with the state playing a secondary overseeing role. In the UK however, the most significant actor is arguably WRAP. WRAP was established by government as a not‐for‐profit company to promote sustainable resource use, and it was later registered as a charity.^[^ ^56^, ^78^ ^]^ It is WRAP, not a government department or regulator, that administers the Courtauld Commitments and undertakes several associated quasi‐regulatory/state activities. These include convening working groups, producing written guidance, and distributing public funding. WRAP is widely regarded as “instrumental” in raising the profile of food waste,^[^ ^56^, ^76^ ^]^ with “unanimity” as to the critical role WRAP has played as a trusted and neutral intermediary.^[^ ^79^, ^80^ ^]^

This approach does nonetheless emphasize the role of private actors (rather than the state) in preventing food waste via the “business case”, with clear hallmarks of a markets approach. For example, a central strand of WRAP's activity is working with major retailers to prevent food waste in households, where much food is wasted.^[^ ^71^ ^]^ This reflects government policy seeking to enroll retailers as “surrogate regulators” of food waste,^[^ ^70^ ^]^ see, e.g.,^[^ ^81^ ^]^ with retailers expected (but not required) to take the lead in preventing food waste upstream in the supply chain, supported by a range of guides and processes provided by WRAP.^[^ ^72^, ^76^ ^]^ While levels of retail waste are relatively low,^[^ ^56^ ^]^ government policy increasingly encourages third sector organizations to redistribute this surplus, and in a markets fashion, instructs civil society organizations to “think like a business” (i.e., that non‐profit organizations should act according to a market logic).^[^ ^74^ ^]^

Seeking to mobilize consumer behavior around household food waste prevention has also been central. However, the construction of food waste as an “end‐of‐pipe” consumer problem has been criticized as disproportionately “blaming” individuals for a structural problem while also distracting from the need to prevent waste upstream,^[^ ^82^, ^83^, ^84^ ^]^ particularly on farms.^[^ ^56^, ^58^ ^]^

#### 3.3.4 Focus Areas of Food Waste Governance Architecture

While England is *the* exemplar of the voluntary approach to food waste prevention, with considerable energy diverted to surplus food redistribution together with preventing household food waste through soft initiatives such as information campaigns,^[^ ^56^, ^85^ ^]^ the state is more interventionist with respect to waste management activities such as treatment and disposal. Nonetheless, this is done in a manner consistent with the markets approach, with pricing mechanisms—such as a landfill tax and subsidies for anaerobic digestion (turning food waste in to biofuels)—supporting landfill diversion.^[^ ^70^, ^86^ ^]^ Evidence suggests these incentives hamper surplus food redistribution efforts.^[^ ^87^, ^88^, ^89^ ^]^ While levels of surplus food redistribution have increased through grant funding to the third sector,^[^ ^90^ ^]^ measures more explicitly tackling overproduction are yet to materialize.^[^ ^58^, ^91^ ^]^

### 3.4 Norway

#### 3.4.1 Historical Development

Food waste became a policy issue in Norway after 2008, when the food‐retailing sector identified it as a pressing problem.^[^ ^10^ ^]^ However, prior to 2010, when the ForMat project, the first systematic attempt to tackle food waste, was launched, no national statistics on food waste existed.^[^ ^9^, ^92^ ^]^ Since 2012, ForMat was administered by Matvett AS, a non‐profit multi‐stakeholder hub, working with the business sector, five ministries, and the research institute NORSUS (previously known as Østfoldforskning). In 2015, food sector companies signed an Agreement of Intent to reduce food waste,^[^ ^93^ ^]^ followed by the Industry Agreement on the reduction of food waste signed in 2017 by a dozen industry organizations and several ministries.^[^ ^94^ ^]^ At the time of writing, just over 100 companies have joined the voluntary agreement. NGOs have also been increasingly active in shaping food waste governance. The organization Future in our Hands started launching educational campaigns and pressing for increased industry ambition in 2012. Another NGO, Food Banks Norway, started working on surplus food redistribution.^[^ ^9^ ^]^ Companies can request value added tax refunds for donated food products, but the same rule also applies to all discarded food.

#### 3.4.2 Steering Modes

Current food waste governance in Norway is non‐hierarchical, which is very much in line with Norway's public policy traditions that see a strong commitment to subsidiarity, decentralized decision making, local governance, and a strong collaboration between civil society, private business and labor organizations, with state institutions in an auxiliary role.^[^ ^95^ ^]^

The examples of France and Italy opened the space for policy learning. As of mid‐2024, there are no penalties for not meeting the targets and the Industry Agreement can be terminated with one month's notice. Some companies struggle to reach the Agreement's targets, while others met them long before or introduced their own thresholds. For almost ten years the “shadow of hierarchy”, that is the looming threat of governmental regulation, was cast over the Norwegian food waste governance. It was anticipated that, if the Industry Agreement does not deliver or appears to focus only on low hanging fruit, the state will likely step in with mandated self‐regulation or direct regulation.^[^ ^9^ ^]^ If, on the contrary, the Agreement proved a success, the government might scale up the Agreement's provisions to cover the entire industry. Following the September 2021 general election, the incoming Labor‐Centre government announced its dedication to a Food Waste Law.^[^ ^96^ ^]^ Finally in early 2023 a special committee got the mandate to find measures that can halve food waste. The committee did not recommend one particular food waste law, but pointed to 35 measures, where some could constitute food waste legislation.^[^ ^97^ ^]^

#### 3.4.3 Key Actors

Norway's approach stands out due to the centrality of the food industry, i.e., the current food waste reduction governance framework, built around the Industry Agreement, relies on private actors.^[^ ^10^ ^]^ Public actors are, however, becoming increasingly active.

At this stage, industry is still the main driver of the process and controls self‐regulation, but outside pressure underlines expectations of particular outcomes for this voluntary process. Here, a central role of Matvett AS can be observed in its role of a secretariat of the special committee. However, the committee is broadly composed of representatives from the food value chain, the municipal sector, consumer, and environmental organizations as well as research environments.

State agencies are taking over some food waste reduction tasks, including a system for industry reporting, building national statistics, reporting to the EU, producing reports, influencing consumer behavior, supporting donation, and involving other public sector actors.^[^ ^94^ ^]^ At the same time, the state has been withdrawing financial support for industrial food waste reduction work and seemingly sees this as the burden for the industry.

#### 3.4.4 Focus Areas of Food Waste Governance Architecture

The Industry Agreement lists eighteen measures that need to be undertaken by industry and authorities. Significant emphasis is put on data gathering and coordination so most of the measures listed cannot be classified according to the level of intervention. It can be noted that the agreement shows some general directions of food waste reduction work and responsibilities. It can therefore give actors flexibility in shaping reduction measures, but can also easily hamper ambition and promote inaction. The measures listed are rarely concrete. Several are dedicated to prevention, re‐use, and recovery and they imply collaboration (food industry and authorities, food industry and NGOs, authorities and consumers, authorities, food industry and NGOs). Interestingly, prevention is listed in the consumer context, while there is an emphasis on re‐use and donations for the industry. In practice, the industry elaborated its own pyramid where preference is given to selling at full price, followed by discount selling and donation.^[^ ^92^ ^]^ More ambitious measures dedicated to “strong prevention” by tackling overproduction, food waste on farms, or systemic failures are still lacking and the new recommendations largely rely on voluntarism. They suggest among others that the Industry Agreement needs to be strengthened and expanded and businesses should carry out risk assessments and implement tailored measures to avoid food waste in their own business and across the value chain, including toward the consumer.

## 4 Discussion

By exploring the case studies focusing on two dimensions of governance architectures (steering modes and actors), we reveal both distinguishing features and similarities that in turn challenge assumptions around food waste reduction governance. Our analysis shows that the typical one‐dimensional distinction between “state driven” on the one hand, and “voluntary” approaches to food waste governance on the other, can be challenged.

**Figure 2:**
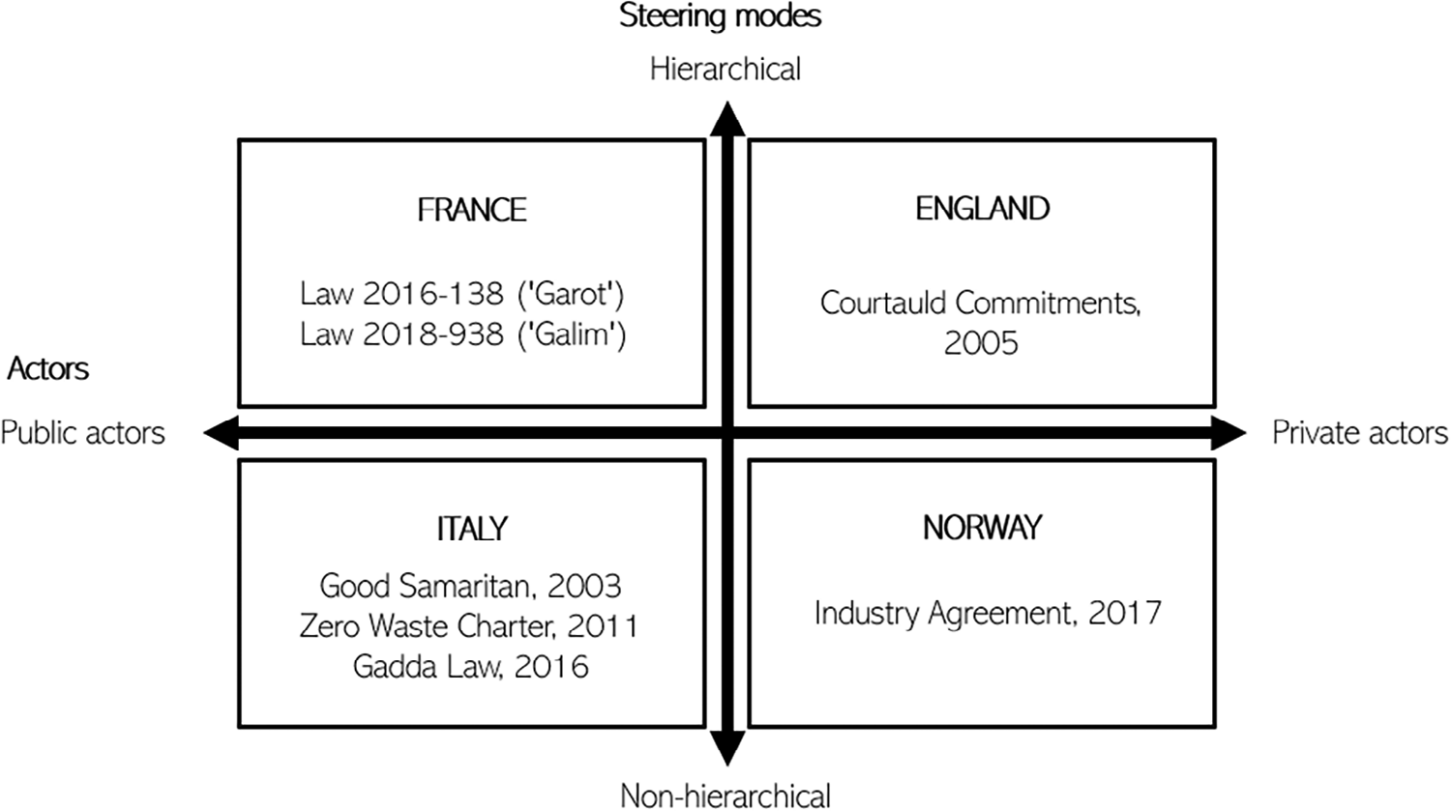
Country cases, governance approaches, and key policy documents (own elaboration).

Contrary to the law versus no law distinction, which would see France and Italy in one category, we find that Italy and Norway have adopted less hierarchical governance approaches than France and England (see **Figure** **2**). However, while the French and English approaches to food waste are typically presented as diametrically opposed (legislative v. voluntary), our analysis reveals this as a false dichotomy. For example, France's legislative “top‐down” approach bears hallmarks of markets more obviously associated with England's voluntary “business case” approach. While France has adopted a circular economy approach to redistribution, reconfiguring the sector to attract private parties, England has offered funding directly to third sector charities. However, the French approach appears to have some different focus areas, with tax incentives applying to food donation. This contrasts with England's marketized approach to waste *management* through subsidies for anaerobic digestion that in turn have harmed redistribution. While the circular economy approach in England has been criticized as promoting a narrative of “the more waste, the better”,^[^ ^87^, ^98^ ^]^ for the reasons given above, the French approach might be criticized for promoting “the more surplus, the better” (**Table** **1**).

**Table 1:** Food waste governance architectures: Summary of comparative case study analysis.

Binding law	France	England	Norway	Italy
	Yes	No	No	Yes
Key elements of food waste reduction policy	Extensive use of food waste legislation and regulation	Outsourcing the administration of voluntary efforts to WRAP	Industry Agreement on the reduction of food waste	National Food Loss and Waste Prevention Plan, PINPAS, National Table for Combating Food Waste, laws for food donation
	Hierarchical		Non‐hierarchical	
Steering modes	Hierarchies – a “hard” legislative approach via the state	Clear privatization of governance and hierarchy through markets	Non‐hierarchical communities emerging around self‐regulation initiatives	Non‐hierarchical public‐private networks across scales, with strong involvement of municipal authorities
Key actors	State actors, market actors, big initial emphasis on retailers	WRAP through the Courtauld Commitments	Matvett AS and food industry companies, ministries, NGOs	National authorities, industry, NGOs, important role of municipal authorities
Focus of governance architecture	re‐use/ redistribution of surplus food	surplus food redistribution together with preventing household food waste through soft initiatives	work with the food industry, support for food redistribution	special emphasis on surplus donations
General trends	legislative turn (laws and legislative “threats”) focus on “low hanging fruits”, a neoliberal paradigm (large emphasis on redistribution and new business models)			

An analysis of actors is also revealing. England for example has “outsourced” food waste administration to WRAP, in receipt of significant (though dwindling) public funding that in turn provides financial support to third sector redistribution organizations. WRAP provides a quasi‐public institutional presence that does not appear to be mirrored in France or Italy, where a clearer role for public actors might be expected. Interestingly in Italy, it is the cities (i.e., municipal level authorities) that show the ambition to become food waste governance hubs (e.g., Milan).^[^ ^63^ ^]^

However, WRAP is under‐conceptualized and under‐researched, both from a governance perspective and as a food waste intervention *in and of itself*. A comparison between WRAP in the UK and Matvett in Norway is also instructive of further research that might be carried out in this area, raising related questions around accountability and legitimacy. Both Matvett and WRAP were fundamental in opening the public debate on food waste and are national “hubs” of expertise and influence. However, they are both potentially constrained by their funding settlements. Most of WRAP's income is from UK governments^[^ ^78^, ^90^ ^]^ and is potentially limited in its ability to engage in a robust public dialogue with government about food waste policy. Matvett in turn may be limited in growing further and in its ability to question levels of industry ambition given it is heavily reliant on the Norwegian food industry for funding.

While there is emphasis on retailers in France, England, and Norway, the construction of retailer responsibility for food waste appears to differ. For example, under the French Garot Law, the responsibility of retailers is primarily for food waste within their own operations. In contrast, retailers in the UK are increasingly mobilized as surrogate regulators of waste elsewhere in the supply chain, and in Norway, some retailers are also starting to raise consumer awareness on food waste. While the ability of retailers to drive meaningful change in the absence of legislative measures has been doubted,^[^ ^58^, ^88^, ^99^, ^100^ ^]^ this broader conceptualisation of their role in food waste prevention is more in‐keeping with calls for distributing responsibility for food waste *along* the supply chain.^[^ ^80^ ^]^

At the same time, while household waste levels have understandably driven an interest in changing consumer behavior, there is limited activity across all four cases to tackle “strong” prevention with problems related to overproduction, including on farms or general product abundance. Arguably, this relates to the structural limitations of housing food waste law and policy within frameworks on waste, as opposed to food and agriculture,^[^ ^87^ ^]^ confirming Messner's “prevention paradox” that describes a proclaimed focus on preventing and a working focus on managing food waste.^[^ ^101^ ^]^

Emphasis is increasingly placed, particularly in the grey literature, on legislative support for food redistribution, especially “Good Samaritan” Laws and tax incentives.^[^ ^102^ ^]^ The Norwegian case shows that even when the state offers tax exemptions for donated food, the systemic approach fails, as the same scheme is used for food thrown away, treating food waste prevention and food waste management equally. While Italy's Good Samaritan Law has been held up an example of best practice,^[^ ^103^ ^]^ seeking to encourage for‐profit private actors to donate surplus food through liability protections (see, e.g.,^[^ ^104^, ^105^ ^]^) and publicly‐funded tax breaks^[^ ^52^, ^106^ ^]^ is not uncontroversial. Similarly, while the EU (and England and Norway) shift toward legal obligations to measure and report on food waste, it should be remembered that measurement itself is no guarantee of preventative actions.^[^ ^107^ ^]^

## 5 Conclusion

Since the governance of food waste is a relatively new field for policymakers, policy implementation and data availability for national contexts is, comparatively in size and scope, severely limited.^[^ ^5^ ^]^ Our analysis of four leading food waste jurisdictions—France, England, Norway, and Italy—unpacks the hidden realities obscured by the simplistic division of legislative versus voluntary approaches. For example, while France is typically presented as the archetypically legislative food waste regime, it has many markets governance features, and Italy's legislative interventions are rooted in non‐hierarchal *voluntarism*.

While we were to some extent able to place the case studies within the governance approaches matrix based on Hall 2011,^[^ ^37^ ^]^ which adds nuance to the way emergent food waste reduction governance architectures can be categorized and divided (see Figure 2), there are also several uniting general tendencies visible in all studied cases.^[^ ^108^ ^]^

First, there is some evidence of a “legislative turn”. For example, in the hitherto “non‐intervention” approaches of England and Norway, policy discussions underway imply a greater role for the state. It is also worth noting that many of the French policy initiatives aimed at providing guidelines and incentives were initially classified as voluntary but later mandated. It can also be noted that the legislative threat is a powerful governance tool for mobilizing the actors in more ambitious food waste prevention and food waste laws may be very diverse in their scope, emphasis and execution. Food waste governance should be seen as an experimentalist policy arena practiced at the national level, and inherently linked to local food production, climate, lifestyles, food habits and norms.^[^ ^10^ ^]^

Second, we note that food waste prevention work starts with picking “low hanging fruits”. Quick effects were initially seen with relatively simple interventions targeting both business actors and consumers. As the ambition (and costs) of food waste prevention begin to bite in the run up to SDG 12.3 deadline, more conflicts emerge, particularly given concerns that powerful actors, especially retailers, have been central in shaping the limited obligations in France^[^ ^42^ ^]^ and England.^[^ ^58^ ^]^ Policymakers need to show more strength enforcing policies against the interests of powerful agents if food waste reduction targets are to be achieved.

Finally, an overall harmonization of responses and convergence in a “neoliberal” paradigm, with redistribution and emphasis on business innovation and finding new profit‐generating activities often pursued as a panacea for the food waste crisis, is highly visible in all studied cases. We note that even community organizations in food waste redistribution start to indirectly contribute to the neoliberal governance model.^[^ ^109^ ^]^

While vested economic interests may present a barrier to the passing of effective laws, care should also be taken to design legislation that tackles the root causes (rather than symptoms) of food waste, including overproduction. This highlights the ongoing European priority for mandatory food waste measurement from farm‐to‐fork *alongside* legislation that targets prevention within the broader context of the food system.

## Conflict of Interest

The authors declare no conflict of interest.

## Data Availability

Data sharing is not applicable to this article as no new data were created or analyzed in this study.
